# (*E*)-1-(2-Amino­phen­yl)-3-(thio­phen-2-yl)prop-2-en-1-one

**DOI:** 10.1107/S1600536813014189

**Published:** 2013-06-08

**Authors:** Suchada Chantrapromma, Pumsak Ruanwas, Nawong Boonnak, Hoong-Kun Fun

**Affiliations:** a Department of Chemistry, Faculty of Science, Prince of Songkla University, Hat-Yai, Songkhla 90112, Thailand; b Faculty of Traditional Thai Medicine, Prince of Songkla University, Hat-Yai, Songkhla 90112, Thailand; c X-ray Crystallography Unit, School of Physics, Universiti Sains Malaysia, 11800 USM, Penang, Malaysia; d Department of Pharmaceutical Chemistry, College of Pharmacy, King Saud University, PO Box 2457, Riyadh 11451, Saudi Arabia

## Abstract

The mol­ecule of the title heteroaryl chalcone derivative, C_13_H_11_NOS, exists in a *trans*-configuaration and is almost planar with a dihedral angle of 3.73 (8)° between the phenyl and thio­phene rings. An intra­molecular N—H⋯O hydrogen bond generates an *S*(6) ring motif. In the crystal, two adjacent mol­ecules are linked into a dimer in an anti-parallel face-to-face manner by a pair of C—H⋯O inter­actions. Neighboring dimers are further linked into chains along the *c-*axis direction by N—H⋯N hydrogen bonds.

## Related literature

For standard bond lengths, see: Allen *et al.* (1987 ▶). For graph-set notation, see: Bernstein *et al.* (1995 ▶). For related structures, see: Fun *et al.* (2011 ▶); Suwunwong *et al.* (2009 ▶). For background to and applications of chalcones, see: Go *et al.* (2005 ▶); Liu *et al.* (2008 ▶); Molyneux (2004 ▶); Nerya *et al.* (2004 ▶); Ni *et al.* (2004 ▶); Shenvi *et al.* (2013 ▶); Suwunwong *et al.* (2011 ▶). For the stability of the temperature controller used in the data collection, see: Cosier & Glazer, (1986 ▶).
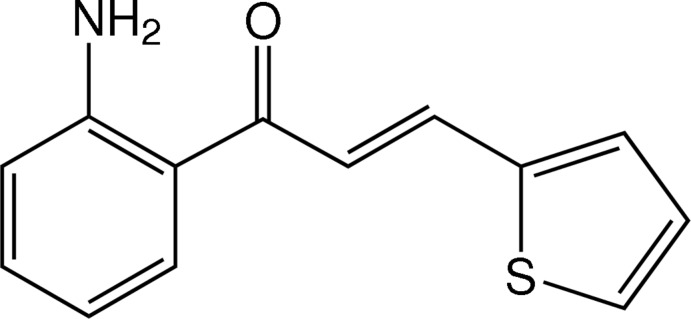


## Experimental

### 

#### Crystal data

C_13_H_11_NOS
*M* *_r_* = 229.30
Monoclinic, 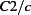

*a* = 24.9335 (4) Å
*b* = 5.0278 (1) Å
*c* = 18.6813 (3) Å
β = 111.151 (1)°
*V* = 2184.13 (7) Å^3^
*Z* = 8
Mo *K*α radiation
μ = 0.27 mm^−1^
*T* = 100 K
0.36 × 0.12 × 0.06 mm

#### Data collection

Bruker APEXII CCD area-detector diffractometer
Absorption correction: multi-scan (*SADABS*; Bruker, 2009 ▶) *T*_min_ = 0.908, *T*_max_ = 0.984
14827 measured reflections
3942 independent reflections
2620 reflections with *I* > 2σ(*I*)
*R*_int_ = 0.036

#### Refinement

*R*[*F* ^2^ > 2σ(*F* ^2^)] = 0.051
*wR*(*F* ^2^) = 0.127
*S* = 1.04
3942 reflections
153 parameters
H atoms treated by a mixture of independent and constrained refinement
Δρ_max_ = 0.39 e Å^−3^
Δρ_min_ = −0.45 e Å^−3^

### 

Data collection: *APEX2* (Bruker, 2009 ▶); cell refinement: *SAINT* (Bruker, 2009 ▶); data reduction: *SAINT*; program(s) used to solve structure: *SHELXTL* (Sheldrick, 2008 ▶); program(s) used to refine structure: *SHELXTL*; molecular graphics: *SHELXTL*; software used to prepare material for publication: *SHELXTL*, *PLATON* (Spek, 2009 ▶), *Mercury* (Macrae *et al.*, 2006 ▶) and *publCIF* (Westrip, 2010 ▶).

## Supplementary Material

Crystal structure: contains datablock(s) global, I. DOI: 10.1107/S1600536813014189/rz5065sup1.cif

Structure factors: contains datablock(s) I. DOI: 10.1107/S1600536813014189/rz5065Isup2.hkl

Supplementary material file. DOI: 10.1107/S1600536813014189/rz5065Isup3.cml

Additional supplementary materials:  crystallographic information; 3D view; checkCIF report

## Figures and Tables

**Table 1:** Hydrogen-bond geometry (Å, °)

*D*—H⋯*A*	*D*—H	H⋯*A*	*D*⋯*A*	*D*—H⋯*A*
N1—H1*N*1⋯O1	0.83 (2)	1.97 (2)	2.6253 (18)	135.6 (19)
N1—H2*N*1⋯N1^i^	0.86 (2)	2.34 (2)	3.184 (2)	169 (2)
C11—H11*A*⋯O1^ii^	0.95	2.56	3.278 (2)	133

Symmetry codes: (i) 
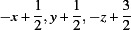
; (ii) 
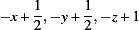
.

**Fig. 1.**
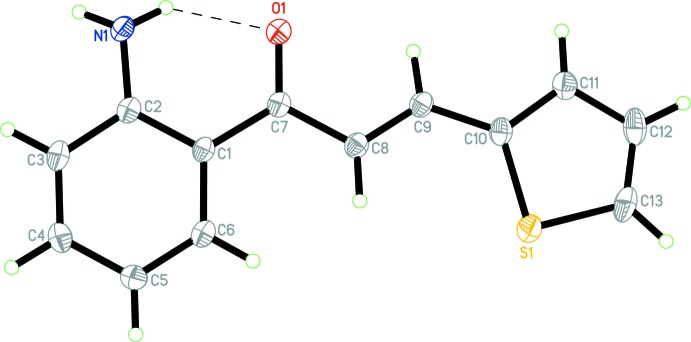
The asymmetric unit of the title compound showing 50% probability displacement ellipsoid. Intramolecular N—H···O hydrogen bond is drawn as dashed line.

**Fig. 2.**
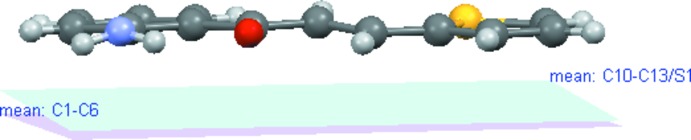
The molecular structure of the title compound showing the approximate planarity of the molecule and the interplanar angle between phenyl and thophene rings.

**Fig. 3.**
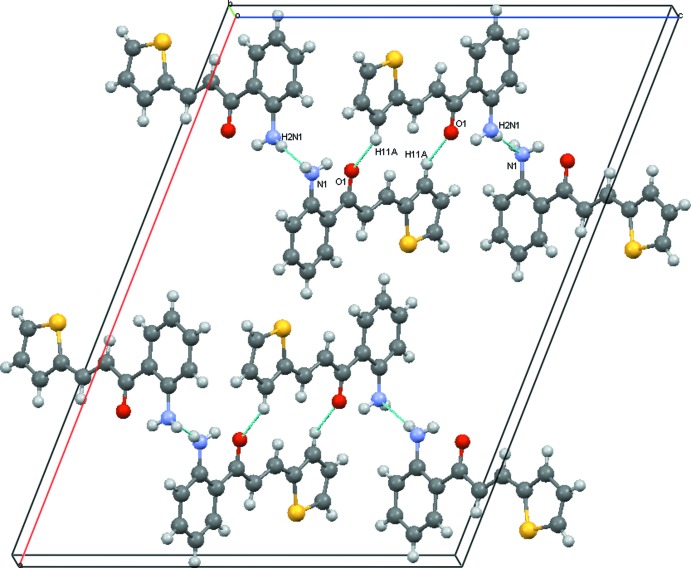
The crystal packing of the title compound viewed along the b axis. Hydrogen bonds are drawn as dashed lines.

**Fig. 4.**
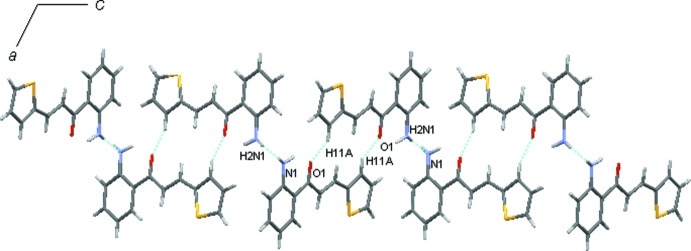
The crystal packing of the title compound, showing a chain of dimers running along the c axis. Hydrogen bonds are drawn as dashed lines.

## supplementary crystallographic information

### Comment

The basic structure of chalcones consists of two aromatic rings bound by an α,β-unsaturated carbonyl group, a unique template associated with various biological activities such as analgesic, anti-inflammatory, antibacterial (Go *et al.*, 2005; Liu *et al.*, 2008; Ni *et al.*, 2004), anticancer and antioxidant (Shenvi *et al.*, 2013) as well as tyrosinase inhibitory (Nerya *et al.*, 2004) and fluorescence (Suwunwong *et al.*, 2011) properties. The title compound (I) was synthesized and studied for antioxidant activity by the DPPH scavenging method (Molyneux, 2004). Our result showed that (I) exhibits a weakly antioxidant activity. It was also tested for antityrosinase activity but found to be inactive. Herein we report the crystal structure of (I).

The molecular structure of (I) exists in a *trans* configuration with respect to the C8═C9 double bond [1.340 (2)°] as indicated by the torsion angle C7–C8–C9–C10 = 179.29 (15)° (Fig. 1). The whole molecule is almost planar, the interplanar angle between phenyl and thiophene rings being 3.73 (8)° (Fig. 2). The propenone unit (C7—C9/O1) is almost planar with the torsion angle O1–C7–C8–C9 = -7.8 (2)°. The mean plane through the propenone bridge makes the dihedral angles of 7.37 (10) and 3.66 (10)° with the phenyl and thiophene rings, respectively. Intramolecular N1—H1N1···O1 hydrogen bond between amino and enone groups (Fig. 1 and Table 1) generates S(6) ring motif (Bernstein *et al.*, 1995). This intramolecular hydrogen bond helps to stabilize the planarity of the structure. However it may result in the prohibition of the α,β-unsaturated carbonyl moiety to be reactive. The bond distances in (I) agree with the literature values (Allen *et al.*, 1987) and are comparable with those observed in related structures (Fun *et al.*, 2011; Suwunwong *et al.*, 2009).

In the crystal packing (Fig. 3), two adjacent molecules are linked in an anti-parallel face-to-face manner into a dimer by a pair of C_thiophene_—H···O interactions and the neighboring dimers are further linked into chains along the *c* axis by N—H···N hydrogen bonds (Fig. 4 and Table 1).

### Experimental

The title compound (I) was prepared by mixing 2-aminoacetophenone (0.40 g, 3 mmol) and 2-thiophenecarboxaldehyde (0.34 g, 3 mmol) in ethanol (30 ml). 30% NaOH aqueous solution (5 ml) was then added and the mixture was stirred at room temperature for 2 hr. The yellow solid formed was filtered and washed with distilled water. Yellow block-shaped single crystals of (I) suitable for *x*-ray structure determination were recrystallized from ethanol by slow evaporation at room temperature over a few weeks. M.p. 407–408 K.

### Refinement

Amino H atoms were located in difference maps and refined isotropically. The remaining H atoms were fixed geometrically and allowed to ride on their parent atoms, with d(C—H) = 0.93 Å for aromatic and 0.98 for CH. The *U*_iso_ values were constrained to be 1.2*U*_eq_ of the carrier atoms. Four outliers (1 5 4, 5 5 2, -1 5 5, -33 3 15) were omitted from the last refinement cycles.

### Crystal data

**Table:**

C_13_H_11_NOS	*F*(000) = 960
*M**_r_* = 229.30	*D*_x_ = 1.395 Mg m^−^^3^
Monoclinic, *C*2/*c*	Melting point = 407–408 K
Hall symbol: -C 2yc	Mo *K*α radiation, λ = 0.71073 Å
*a* = 24.9335 (4) Å	Cell parameters from 3942 reflections
*b* = 5.0278 (1) Å	θ = 1.8–32.5°
*c* = 18.6813 (3) Å	µ = 0.27 mm^−^^1^
β = 111.151 (1)°	*T* = 100 K
*V* = 2184.13 (7) Å^3^	Block, yellow
*Z* = 8	0.36 × 0.12 × 0.06 mm

### Data collection

**Table:**

Bruker APEXII CCD area-detector diffractometer	3942 independent reflections
Radiation source: sealed tube	2620 reflections with *I* > 2σ(*I*)
Graphite monochromator	*R*_int_ = 0.036
φ and ω scans	θ_max_ = 32.5°, θ_min_ = 1.8°
Absorption correction: multi-scan (*SADABS*; Bruker, 2009)	*h* = −37→37
*T*_min_ = 0.908, *T*_max_ = 0.984	*k* = −7→5
14827 measured reflections	*l* = −28→28

### Refinement

**Table:**

Refinement on *F*^2^	Primary atom site location: structure-invariant direct methods
Least-squares matrix: full	Secondary atom site location: difference Fourier map
*R*[*F*^2^ > 2σ(*F*^2^)] = 0.051	Hydrogen site location: inferred from neighbouring sites
*wR*(*F*^2^) = 0.127	H atoms treated by a mixture of independent and constrained refinement
*S* = 1.04	*w* = 1/[σ^2^(*F*_o_^2^) + (0.0488*P*)^2^ + 2.0262*P*] where *P* = (*F*_o_^2^ + 2*F*_c_^2^)/3
3942 reflections	(Δ/σ)_max_ = 0.001
153 parameters	Δρ_max_ = 0.39 e Å^−^^3^
0 restraints	Δρ_min_ = −0.45 e Å^−^^3^

### Special details

**Table:**

Experimental. The crystal was placed in the cold stream of an Oxford Cryosystems Cobra open-flow nitrogen cryostat (Cosier & Glazer, 1986) operating at 100.0 (1) K.
Geometry. All e.s.d.'s (except the e.s.d. in the dihedral angle between two l.s. planes) are estimated using the full covariance matrix. The cell e.s.d.'s are taken into account individually in the estimation of e.s.d.'s in distances, angles and torsion angles; correlations between e.s.d.'s in cell parameters are only used when they are defined by crystal symmetry. An approximate (isotropic) treatment of cell e.s.d.'s is used for estimating e.s.d.'s involving l.s. planes.
Refinement. Refinement of *F*^2^ against ALL reflections. The weighted *R*-factor *wR* and goodness of fit *S* are based on *F*^2^, conventional *R*-factors *R* are based on *F*, with *F* set to zero for negative *F*^2^. The threshold expression of *F*^2^ > σ(*F*^2^) is used only for calculating *R*-factors(gt) *etc*. and is not relevant to the choice of reflections for refinement. *R*-factors based on *F*^2^ are statistically about twice as large as those based on *F*, and *R*- factors based on ALL data will be even larger.

### Fractional atomic coordinates and isotropic or equivalent isotropic displacement parameters (Å^2^)

**Table:**

	*x*	*y*	*z*	*U*_iso_*/*U*_eq_	
S1	0.071728 (19)	−0.24213 (9)	0.38943 (3)	0.02647 (13)	
O1	0.21891 (5)	0.4157 (2)	0.59945 (7)	0.0243 (3)	
N1	0.22281 (6)	0.7977 (3)	0.69701 (8)	0.0196 (3)	
H1N1	0.2382 (8)	0.721 (4)	0.6700 (11)	0.021 (5)*	
H2N1	0.2361 (9)	0.944 (5)	0.7198 (12)	0.037 (6)*	
C1	0.13438 (6)	0.5801 (3)	0.61394 (9)	0.0157 (3)	
C2	0.16405 (7)	0.7756 (3)	0.66901 (9)	0.0161 (3)	
C3	0.13202 (7)	0.9494 (3)	0.69732 (9)	0.0193 (3)	
H3A	0.1516	1.0791	0.7346	0.023*	
C4	0.07321 (7)	0.9354 (3)	0.67233 (10)	0.0210 (3)	
H4A	0.0526	1.0560	0.6919	0.025*	
C5	0.04342 (7)	0.7438 (3)	0.61801 (10)	0.0214 (3)	
H5A	0.0027	0.7337	0.6007	0.026*	
C6	0.07387 (7)	0.5711 (3)	0.59021 (9)	0.0197 (3)	
H6A	0.0535	0.4408	0.5537	0.024*	
C7	0.16602 (7)	0.3989 (3)	0.58064 (9)	0.0166 (3)	
C8	0.13517 (7)	0.1944 (3)	0.52357 (9)	0.0176 (3)	
H8A	0.0955	0.1613	0.5126	0.021*	
C9	0.16398 (7)	0.0568 (3)	0.48761 (9)	0.0181 (3)	
H9A	0.2034	0.1021	0.5010	0.022*	
C10	0.14298 (7)	−0.1511 (3)	0.43121 (9)	0.0186 (3)	
C11	0.17609 (7)	−0.3053 (3)	0.40209 (10)	0.0210 (3)	
H11A	0.2166	−0.2864	0.4176	0.025*	
C12	0.14450 (8)	−0.4928 (4)	0.34746 (10)	0.0267 (4)	
H12A	0.1613	−0.6149	0.3228	0.032*	
C13	0.08760 (8)	−0.4801 (4)	0.33398 (10)	0.0275 (4)	
H13A	0.0596	−0.5897	0.2981	0.033*	

### Atomic displacement parameters (Å^2^)

**Table:**

	*U*^11^	*U*^22^	*U*^33^	*U*^12^	*U*^13^	*U*^23^
S1	0.0228 (2)	0.0244 (2)	0.0278 (2)	0.00019 (17)	0.00385 (17)	−0.00763 (19)
O1	0.0191 (6)	0.0249 (6)	0.0278 (7)	−0.0020 (5)	0.0071 (5)	−0.0082 (5)
N1	0.0185 (7)	0.0191 (7)	0.0187 (7)	−0.0015 (5)	0.0035 (6)	−0.0039 (6)
C1	0.0186 (7)	0.0133 (6)	0.0144 (7)	−0.0012 (6)	0.0050 (6)	0.0004 (6)
C2	0.0203 (7)	0.0143 (6)	0.0125 (7)	−0.0003 (6)	0.0043 (6)	0.0027 (6)
C3	0.0265 (8)	0.0151 (7)	0.0144 (7)	−0.0006 (6)	0.0054 (6)	−0.0005 (6)
C4	0.0265 (8)	0.0185 (7)	0.0195 (8)	0.0037 (6)	0.0100 (7)	0.0003 (6)
C5	0.0180 (7)	0.0224 (7)	0.0241 (8)	0.0004 (7)	0.0078 (6)	−0.0015 (7)
C6	0.0218 (8)	0.0172 (7)	0.0186 (8)	−0.0024 (6)	0.0056 (6)	−0.0020 (6)
C7	0.0195 (8)	0.0144 (7)	0.0151 (7)	−0.0004 (6)	0.0055 (6)	0.0012 (6)
C8	0.0190 (7)	0.0149 (7)	0.0180 (7)	−0.0012 (6)	0.0057 (6)	0.0003 (6)
C9	0.0201 (8)	0.0163 (7)	0.0172 (7)	−0.0020 (6)	0.0060 (6)	0.0001 (6)
C10	0.0241 (8)	0.0151 (7)	0.0179 (8)	−0.0014 (6)	0.0090 (6)	0.0003 (6)
C11	0.0252 (8)	0.0202 (8)	0.0228 (8)	−0.0027 (6)	0.0148 (7)	−0.0014 (6)
C12	0.0443 (11)	0.0195 (8)	0.0221 (9)	−0.0013 (8)	0.0191 (8)	−0.0029 (7)
C13	0.0384 (10)	0.0205 (8)	0.0192 (8)	−0.0038 (7)	0.0050 (7)	−0.0055 (7)

### Geometric parameters (Å, º)

**Table:**

S1—C13	1.7195 (19)	C5—C6	1.373 (2)
S1—C10	1.7245 (17)	C5—H5A	0.9500
O1—C7	1.2392 (19)	C6—H6A	0.9500
N1—C2	1.371 (2)	C7—C8	1.481 (2)
N1—H1N1	0.83 (2)	C8—C9	1.340 (2)
N1—H2N1	0.86 (2)	C8—H8A	0.9500
C1—C6	1.412 (2)	C9—C10	1.442 (2)
C1—C2	1.422 (2)	C9—H9A	0.9500
C1—C7	1.481 (2)	C10—C11	1.380 (2)
C2—C3	1.409 (2)	C11—C12	1.404 (2)
C3—C4	1.371 (2)	C11—H11A	0.9500
C3—H3A	0.9500	C12—C13	1.350 (3)
C4—C5	1.402 (2)	C12—H12A	0.9500
C4—H4A	0.9500	C13—H13A	0.9500
			
C13—S1—C10	91.94 (9)	O1—C7—C1	120.84 (14)
C2—N1—H1N1	113.2 (13)	O1—C7—C8	118.34 (14)
C2—N1—H2N1	115.3 (14)	C1—C7—C8	120.82 (14)
H1N1—N1—H2N1	122 (2)	C9—C8—C7	119.10 (15)
C6—C1—C2	117.86 (14)	C9—C8—H8A	120.4
C6—C1—C7	121.29 (14)	C7—C8—H8A	120.4
C2—C1—C7	120.81 (14)	C8—C9—C10	128.46 (15)
N1—C2—C3	118.61 (14)	C8—C9—H9A	115.8
N1—C2—C1	122.50 (14)	C10—C9—H9A	115.8
C3—C2—C1	118.88 (14)	C11—C10—C9	125.77 (15)
C4—C3—C2	121.45 (15)	C11—C10—S1	109.74 (12)
C4—C3—H3A	119.3	C9—C10—S1	124.49 (12)
C2—C3—H3A	119.3	C10—C11—C12	113.91 (16)
C3—C4—C5	120.26 (15)	C10—C11—H11A	123.0
C3—C4—H4A	119.9	C12—C11—H11A	123.0
C5—C4—H4A	119.9	C13—C12—C11	112.33 (16)
C6—C5—C4	119.19 (15)	C13—C12—H12A	123.8
C6—C5—H5A	120.4	C11—C12—H12A	123.8
C4—C5—H5A	120.4	C12—C13—S1	112.06 (13)
C5—C6—C1	122.36 (15)	C12—C13—H13A	124.0
C5—C6—H6A	118.8	S1—C13—H13A	124.0
C1—C6—H6A	118.8		
			
C6—C1—C2—N1	178.64 (14)	C2—C1—C7—C8	179.69 (14)
C7—C1—C2—N1	−3.6 (2)	O1—C7—C8—C9	−7.8 (2)
C6—C1—C2—C3	0.2 (2)	C1—C7—C8—C9	171.71 (14)
C7—C1—C2—C3	177.97 (14)	C7—C8—C9—C10	179.29 (15)
N1—C2—C3—C4	−179.23 (15)	C8—C9—C10—C11	−172.66 (17)
C1—C2—C3—C4	−0.7 (2)	C8—C9—C10—S1	7.8 (3)
C2—C3—C4—C5	0.7 (2)	C13—S1—C10—C11	−0.44 (13)
C3—C4—C5—C6	−0.1 (3)	C13—S1—C10—C9	179.13 (15)
C4—C5—C6—C1	−0.5 (3)	C9—C10—C11—C12	−179.72 (16)
C2—C1—C6—C5	0.4 (2)	S1—C10—C11—C12	−0.15 (18)
C7—C1—C6—C5	−177.38 (15)	C10—C11—C12—C13	0.9 (2)
C6—C1—C7—O1	176.95 (15)	C11—C12—C13—S1	−1.2 (2)
C2—C1—C7—O1	−0.8 (2)	C10—S1—C13—C12	0.96 (15)
C6—C1—C7—C8	−2.6 (2)		

### Hydrogen-bond geometry (Å, º)

**Table:**

*D*—H···*A*	*D*—H	H···*A*	*D*···*A*	*D*—H···*A*
N1—H1*N*1···O1	0.83 (2)	1.97 (2)	2.6253 (18)	135.6 (19)
N1—H2*N*1···N1^i^	0.86 (2)	2.34 (2)	3.184 (2)	169 (2)
C11—H11*A*···O1^ii^	0.95	2.56	3.278 (2)	133

Symmetry codes: (i) −*x*+1/2, *y*+1/2, −*z*+3/2; (ii) −*x*+1/2, −*y*+1/2, −*z*+1.
